# Identifying and addressing challenges to antimicrobial use surveillance in the human health sector in low- and middle-income countries: experiences and lessons learned from Tanzania and Uganda

**DOI:** 10.1186/s13756-023-01213-3

**Published:** 2023-02-09

**Authors:** Reuben Kiggundu, Edgar Lusaya, Jeremiah Seni, J. P. Waswa, Francis Kakooza, Dinah Tjipura, Kate Kikule, Cecilia Muiva, Mohan P. Joshi, Andy Stergachis, Freddy Eric Kitutu, Niranjan Konduri

**Affiliations:** 1 USAID Medicines, Technologies, and Pharmaceutical Services (MTaPS) Program, Management Sciences for Health, Kampala, Uganda; 2 USAID Medicines, Technologies, and Pharmaceutical Services (MTaPS) Program, Management Sciences for Health, Dar Es Salaam, Tanzania; 3 Weill Bugando School of Medicine, Catholic University of Health and Allied Sciences, Mwanza, Tanzania; 4 Infectious Diseases Institute, Makerere University College of Health Sciences, Kampala, Uganda; 5 USAID Medicines, Technologies, and Pharmaceutical Services (MTaPS) Program, Management Sciences for Health, Arlington, VA 22203 USA; 6 USAID Medicines, Technologies, and Pharmaceutical Services (MTaPS) Program, Management Sciences for Health, Nairobi, Kenya; 7 Department of Pharmacy, School of Pharmacy, University of Washington, Seattle, WA 98105 USA; 8 Department of Global Health, School of Public Health, University of Washington, Seattle, WA 98105 USA; 9 Sustainable Pharmaceutical Systems (SPS) Unit, Pharmacy Department, Makerere University School of Health Sciences, P.O. Box 10217, Kampala, Uganda

**Keywords:** Antimicrobial resistance, Antimicrobial use surveillance, Capacity building, Health system, Africa, Global health security, Point prevalence survey, Tanzania, Uganda

## Abstract

**Background:**

Antimicrobial resistance (AMR) is a global health security threat and is associated with increased morbidity and mortality. One of the key drivers of AMR is the inappropriate use of antibiotics. A key component of improving antibiotic use is conducting antimicrobial use (AMU) surveillance.

**Methods:**

USAID Medicines Technologies and Pharmaceutical Services Program has supported the implementation of antimicrobial stewardship activities, including setting up systems for AMU surveillance in Tanzania and Uganda. Results from both countries have been previously published. However, additional implementation experience and lessons learned from addressing challenges to AMU surveillance have not been previously published and are the subject of this narrative article.

**Results:**

The team identified challenges including poor quality data, low digitalization of tools, and inadequate resources including both financial and human resources. To address these gaps, the Program has supported the use of continuous quality improvement approaches addressing gaps in skills, providing tools, and developing guidelines to fill policy gaps in AMU surveillance. Recommendations to fill these gaps, based on the Potter and Brough systematic capacity building model have been proposed.

**Conclusions:**

Strengthening AMU surveillance through using a capacity-building approach will fill gaps and strengthen efforts for AMR control in both countries.

## Background

Antimicrobial resistance (AMR) is a sustainable development challenge and global health security threat. It threatens progress toward several sustainable development goals including health. [1, 2]. In 2019, AMR was estimated to directly cause an estimated 1.27million deaths, with a further 4.95 indirect deaths [1] AMR threatens global development initiatives including efforts to control infectious diseases [3]. Multiple efforts to control AMR are currently ongoing [4–7], including the Global Action Plan which proposes 5 strategic objectives, including optimizing the use of antimicrobial agents [5]. Antimicrobial Use Surveillance (AMU) is a component of the optimization of the use of antimicrobial agents.

Inappropriate use of antibiotics is a major driver of AMR and can be observed in many forms but not limited to: wrong dose, wrong indication, incomplete dosage, inappropriate prescribing, and poor disposal of medical waste [8]. It is therefore imperative that we develop systems for AMU [9]. In addition to understanding how antibiotics are used, AMU surveillance can inform the implementation of the WHO Access, Watch, and Reserve (AWaRe) [10] categorization of antibiotics and the WHO Global Antimicrobial Resistance Surveillance System (GLASS) [11].

The WHO’s International Health Regulations (IHR) Benchmark 3.4 on optimizing use of antimicrobials recommended that member states monitor antimicrobial use, among other antimicrobial stewardship (AMS) activities, in designated health facilities to reach level 3 (“developed capacity”) with progression to level 4 (“demonstrated capacity”) and finally to level 5 (“sustainable capacity”) [12]. A recent WHO report found that most low and middle-income countries (LMICs) did not have systems for AMU surveillance [13]. In several countries, available data is fragmented or available as “snapshot” research. Thus there is no comprehensive reflection of AMU surveillance and factors that influence it [14], hence the need for capacity building in this area.

### The context

The Medicines, Technologies and Pharmaceutical Services Program (henceforth called “the Program”) is a five-year Program led by Management Sciences for Health and funded by the United States Agency for International Development [15]. The Program uses a One Health approach to build AMR control capacities in 13 LMICs. Our work focuses on strengthening multisectoral collaboration, infection prevention and control and AMS and is guided by a country’s AMR National Action Plan (NAP) and WHO Benchmarks for IHR [12]. Under AMS, the Program is building systems for AMU surveillance through provision of technical assistance to selected countries. In Tanzania and Uganda, the Program works with in-country entities such as the Ministry of Health, the Multisectoral Coordinating Committee and Technical Working Groups (TWG), health facilities, professional and regulatory bodies, academic institutions such as Muhimbili University of Allied and Health Sciences and Catholic University of Allied and Health Sciences in Tanzania, Makerere University in Uganda, and the University of Washington as a global learning partner. The Program uses a multi-pronged capacity building approach through training, provision of tools and guidelines, on-site mentorship, and supportive supervision at the health facility level. The Program works in 10 and 13 hospitals Tanzania and Uganda, respectively and AMU surveillance findings from these were recently published [16, 17]. More work on AMU surveillance from both countries has also been published [18–20].

Despite these efforts, gaps in implementing and sustaining AMU surveillance remain. In this context, based on our experiences, we describe current challenges and propose recommendations for improving AMU surveillance in Tanzania and Uganda and by extension could be used by other LMICs.

## Challenges facing AMU surveillance in Tanzania and Uganda

Although efforts to strengthen AMU surveillance are ongoing, challenges remain as described below.

### Inadequate data systems including sub-optimal data sources and low digitalization

The capacity to collect and report high-quality data is crucial for successful AMU surveillance. First, in both countries, ineligible handwriting, missing patient files, incomplete medical records, e.g., lack of diagnosis or prescription notes were a challenge during data collection. Second, although Health Management Information System (HMIS) tools are available in both countries, AMU surveillance data collection indicators have not been integrated into these tools, hence the need for introduction of revised tools. This negatively affects the interoperability of data systems and leads to additional strain on the available resources. In both countries, the AMU tool and data sources were manual which increased the level of effort and raised the risks of error during data extraction. Third, there is limited use of standard coding of disease in both countries, with wide variation in nomenclature of diagnosis. For example, while some clinicians wrote the diagnosis as lower respiratory tract infection, others wrote pneumonia. Another common cause of discrepancy was urinary tract infection versus the use of other terminologies like pyelonephritis or cystitis. This created discrepancy in the indication for use of antibiotics both within hospitals, between hospitals and between countries. Lastly, antibiotic prescriptions obtained from outside the hospital dispensing systems could not be verified since prescriptions were not available in the hospital records. With a high stockout of essential medicines in LMICs, improving data management for medicines obtained outside the hospital pharmacy system is essential to understanding hospital antibiotic use pathways [21].

### Lack of appropriate tools for AMU surveillance

As highlighted from AMU surveillance reports published from both countries, there is variability of methodologies [18, 20, 22]. However, the Program’s experience is based on the use of the WHO Point Prevalence Survey methodology [23]. Although the tool has been developed in the context of LMICs, data on some of the variables was not readily available in the medical record system of both countries. For example, data on whether the patient is having a nasogastric tube or urinary catheter is not normally captured in patient files and the data collectors had to examine patients to confirm if or not patients had these in place. Similarly, data on other patient variables like human immunodeficiency virus status, tuberculosis, antiretroviral therapy, CD4 count, McCabe score and, malnutrition status is not collected in a standard way, with physician preference and practices determining if this data is collected and recorded in the patient chart as part of the clinical notes. To avoid missing data, the research assistants had to search for additional data sources, leading to increased level of effort. Lastly, despite the high burden of disease attended to in hospital outpatient departments (OPDs), the available WHO AMU surveillance tool is only applicable in the inpatient department. The Uganda Health Sector Performance Report of 2019/2020 showed a cumulative attendance of 49,995,720 cases and 2,069,310 cases in the OPD and inpatient wards respectively [24]. The current inability to collect OPD AMU data creates gaps in data and may bias AMS interventions, leaving OPDs behind. Tools for AMU surveillance in OPDs have been developed for use in other countries and should be adopted for use in LMICs [25].

### Limited AMS and AMU surveillance technical expertise in the health facilities

Conducting AMU surveillance requires a skilled workforce, with multidisciplinary skills, including epidemiological, clinical, and pharmaceutical knowledge that have expertise in the surveillance methodology. For example, in our experience, the interpretation of diagnosis and indication required a medical doctor or nurse with familiarity in interpreting indications to be part of the team. On the other hand, classification of antibiotics required a pharmacy technician or pharmacist. The Program set up multidisciplinary teams, supported by our staff, national level experts and facility-based health workers to conduct the AMU surveillance, overcoming these challenges in the process. Most hospitals did not have the technical expertise to constitute a multidisciplinary team with experience in AMU surveillance. As such, the Program provided technical expertise for the activity, with input from in-country academic institutions, Ministries of Health, and individual consultants. The University of Washington provided data analysis and data management support for the Uganda AMU surveillance. Another observation was the absence of adequate structures for AMS in the health facilities. Since AMU surveillance is a component of AMS, it is critical that systems for AMS are present in participating hospitals. The Program is working to address this gap.

### Limited financing for AMU surveillance at all levels

AMU surveillance activities are often not routinely funded through hospital budgets nor through a formal budget line item in Ministries of Health. Consequently, the program fully funded the activities. Table 1 summarizes the costs of the AMU surveillance in both countries. We intentionally share the costs knowing that it is not found in the literature. Tanzania is almost four times the geographical size of Uganda and our intervention hospitals in Tanzania are geographically dispersed across regions compared to Uganda. We found the costs for AMU surveillance variable between the two countries. The costs could be higher or lower when applied to the context of hospitals in LMICs. However, if capacity is built at the hospital level, these costs could be significantly lowered. For example, the hospitals do not have to incur costs on travel, lodging and a workshop to train data collectors if capacity is built inhouse. In Uganda, hospitals are currently using the Primary Health Care fund to run their AMR control activities, in addition to using the same funds to support other related activities like community outreach, water sanitation and hygiene and health promotion. However, these funds are inadequate, and gaps exist in financing AMR control activities [26].

**Table 1:** Recommendations for systematic capacity building for AMU surveillance in Tanzania and Uganda *

	National level recommendation	Health facility recommendation	Expense	Cost of PPS for antibiotic use in 6 Tanzania Hospitals (USD)	Cost of PPS for antibiotic use in 13 Uganda hospitals (USD)
Structure, Systems, and roles	Strengthen AMU surveillance of the AMS TWC	Hospital AMS teams			
	National plan	Hospital AMU plans	Training data collectors		
	Strengthen the implementation of policies and regulations on antibiotic use	Appoint hospital committees	Participants per diems, meals, transportation	6555	2493
	Standard operating procedures	Routine monitoring of antibiotic use to be part of antibiotic stewardship program			
	Advocacy		Data collection activities		
	Identify resources				
	Partner engagement		Per diems & accommodation	11,474	5,342
	Private sector engagement		Transportation	11,474	5,342
Staff and Infrastructure	National plan for AMR education, including both pre-service and in-service trainings	Set up hospital AMS programs	Stationery	421	1,521
		Hospital AMS teams set up			
		Implement AMS quality improvement projects			
Skills	Enforcement of mandatory CPDs on AMR for annual licensing	Integrated training	Data analysis (consultant)	10,530	10,000
	Strengthen accreditation and licensing	Create AMR awareness			
Tools	Standardization of tools	Electronic tools			
	Automation of tools	Avail data on AMS			
	Aligning tools with existing HMIS	Develop tools for data collection in the outpatient departments			
	Systems for data sharing		Total	38,838	24,945

## Recommendations for addressing AMU surveillance challenges in Tanzania and Uganda

Despite these challenges, some solutions are proposed for consideration to build a sustainable structure for AMU surveillance in both countries (Table 1). We apply the Potter and Brough model of systematic capacity building[27] to make recommendations for specific actions across the board, that if implemented could build sustainable systematic capacity for AMU surveillance. Using this approach could also have a “spillover” effect on other AMR containment efforts in both countries and other LMICs.

### Apply systematic capacity building targeted towards a whole of approach health systems strengthening for AMU surveillance

To build sustainable AMU surveillance, there is need to apply interventions across all the WHO building blocks of a health system [28]. Human resource needs, governance, service delivery, financing, health information systems and medical products components of the health system that apply to AMU surveillance must be addressed. To achieve this, it is important that a systematic approach to capacity building that addresses key structures, systems, roles, staff and infrastructure, skills and tools [27] is adopted and applied to all the building blocks [29]. The Program is using the USAID Pharmaceutical Systems Framework and the WHO Benchmarks to implement key activities that are aimed at building systematic AMU surveillance capacity in both countries [12, 15, 30].

### Use a phased/gradual implementation approach

Our Program’s use of a gradual implementation approach has shown to be successful for similar settings in low resource settings, as recommended in the WHO GLASS manual for early implementation [31]. This approach allows for consideration of local context, national priorities, available resources and has successfully been used by Uganda to build capacity under the GLASS [13]. In Uganda, a starting point could be rejuvenating the appropriate medicines use unit at the Ministry of Health level and following this up by starting AMU surveillance at sentinel surveillance sites as capacity is gradually built (both technical and logistical) at the national level to add more health facilities to the program and later mandate AMU surveillance in representative hospitals in the country. Advanced support could involve digitalization of efforts, linkage or enforcement of legislation and linking data to AMR surveillance. The Program has made contribution to systematic capacity building through implementing priority WHO Benchmark actions for IHR that would lead to an advanced capacity. Examples of WHO Benchmark actions completed with program support include assessment of policies for antibiotic stewardship in both countries, writing of a NAP for AMS and conducting assessment of systems for AMU surveillance in Tanzania and Uganda, respectively.

### Strengthen leadership and governance for AMU surveillance at all levels

Strengthening leadership and governance for AMU surveillance is critical for AMR control [32].

At the national level, an AMU surveillance team under the AMS multisectoral TWG should be appointed and facilitated (technical expertise, capacity building, resource allocation) to enable them to understand and support the implementation of the long-term vision for AMR control. These bodies should take a major role in vertical coordination, upstream with the MSC-AMR body and downstream with facilities and communities. The AMS TWG can catalyze funding advocacy, coordination, research, reporting, dissemination, overall coordination, link AMU surveillance to AMR surveillance and laboratory capacity, and facilitate the use of data for decision-making. As part of strengthening leadership, there is an urgent need for approval of national AMU surveillance plans, which clearly define roles and responsibilities and provides a platform for establishment of a governance structure. The TWGs and AMS teams could catalyze the South-to-South Learning. For example, Tanzania conducted their AMU surveillance before Uganda and as part of capacity building for Uganda, a technical exchange was organized between the Program’s teams where the Tanzania team shared their experiences. The Program supports multisectoral TWGs in both countries; for example facilitating data sharing through quarterly meetings in both countries and publication of a newsletter in Uganda [33]. However, as a next step, there is need to support set up of an AMU surveillance working team and institutionalize this team into government structures. At the health facility level, there is need to establish and strengthen AMS teams as part of the drug and therapeutics committee which will provide leadership for AMU surveillance. The Program has worked with country partners to set-up AMS teams in 6 and 13 hospitals in Tanzania and Uganda respectively and supported the teams through training and mentorship.

### Strengthen the implementation of policies and regulations on antibiotic prescription and use

Non-prescribed antibiotics are known to increase inappropriate use of antibiotics and increase global use and misuse [34], with the highest non-prescription use found in LMICs, at between 19 and 100% in some cases [35]. Coupled with poor adherence to treatment guidelines in Uganda [17], this practice compounds access to unauthorized parallel markets for antibiotics [36], making AMU surveillance more problematic. Uganda has recently assessed policies and regulations on antibiotic stewardship—a key WHO Benchmark activity, with the activity ongoing in Tanzania. There is a need address the identified gaps in relation to AMU surveillance, strengthen implementation of existing regulations on antibiotic utilization and access, over-the-counter non-prescription access of antibiotics in both countries and control of antibiotic use in the veterinary sector. In Tanzania, the Program supported the development of an AMR NAP and adaptation of the WHO AWaRE categorization. This in turn was integrated into the Tanzania Standard Treatment Guidelines and National Essential Medicine List.

### Build stronger data systems with relevant tools in cognizance of the local country context

First, there is need to conduct review of existing HMIS for AMU surveillance and use the findings to inform the development of relevant electronic tools for AMU data collection. The WHO PPS tools should also be digitalized and incorporated into the data collection tools at the health facilities. In Uganda, linking currently available tools on AMU surveillance into existing HMIS tools like the Pharmaceutical Information Portal, Supervision Performance Assessment and Recognition strategy [37] and the District Health Information System- 2 (DHIS-2) should be considered. Similarly, data collection through the DHIS-2 can be strengthened in Tanzania. The WHONET software [38] could be modified to include a module for AMU surveillance in both countries. The WHONET can as well be integrated with the national DHIS2 system. Through integration, challenges of unavailability of data, missing data and poor data quality could be addressed while also creating a system that allows for data sharing at the health facility and the national level. Additionally, consideration could be made for adapting the International Classification of Disease for coding of diagnosis in both countries and other LMICs. Such a system will allow for similar nomenclature of diagnosis, bring clarity on indication of antibiotics, and allow for progress towards a clinical coding surveillance system, which would support systematic surveillance and minimize human resource needs and the costs of surveillance. Lastly, robust systems should be developed to collect data on AMU from the OPDs. In Uganda, the Program has applied the WHO methodology on drug indicator survey to collect data from the OPD [39].

### Support knowledge and skills transfer at all levels of the health care system

It is urgent to build a critical mass of experts to support AMU surveillance at both the national and health facility level through training and mentorship programs in both countries. To overcome the observed lack of technical capacity for AMU surveillance among health workers, a competency-based curriculum on AMS incorporating AMU surveillance, with additional educational interventions like continuous medical education, mentorships, and continuous professional development sessions should be developed for in-service health professionals. This would be in line with the WHO framework on health worker training for AMR [40]. Additionally, important components on AMS and AMU surveillance should be introduced in pre-service curriculum and their implementation supported during houseman years or internship training to provide a foundation for long term learning for AMR. The facility technical experts will support the development of contextualized AMU metrics, monitor activity performance, validate methodologies, and guide operational research. Lastly, there is need to develop a culture of AMS at the health facilities. This can be achieved through implementation of quality improvement plans, training, and mentorship. In Tanzania, the Program is implementing quality improvement plans in 6 hospitals. In Uganda, the Program has cumulatively supported 131 facility-based continuous medical education sessions benefiting 2,152 health workers, 2 continuous professional development sessions and 34 onsite mentorship visits.

## Conclusion

With increasing global efforts to combat AMR, there is a need for comparative data from countries on AMU surveillance. Various methods and approaches to AMU surveillance have been applied in both countries. AMU surveillance programs are in their infancy and there is a need for alignment of approaches, to generate data on a regular basis to inform interventions (for example the 2023 goal of 60% antibiotics used being from the Access category [41]), but also share globally to inform response efforts. The current approaches to AMU surveillance do not allow for systematic data collection that is representative to allow for a comparison of trends of antibiotic use. Through systematic capacity building, health financing bottlenecks should be addressed including defining the government, private sector engagement, reducing donor dependency of the AMR program, while maintaining relevant international collaborations and partnerships. Lastly, data should be used for action to gradually build capacity for AMU surveillance and AMR containment.

Although the surveillance of AMU should be guided by the national AMU surveillance plan, both countries are not implementing these plans. This will be critical to foundation building for the national program, support long-term sustainable capacity building, enable resource mobilization and allocation, define key roles and responsibilities, and define governance mechanism for data collection, management, and sharing while supporting activity implementation at the sub-national levels and health facility level. Along with this, there is a need for agreement on the best methodological approach for data collection, adopting or developing simple tools that are applicable in resource-constrained settings like Uganda and Tanzania. The standardization of existing methodologies in the context of LMICs is also critical to enabling the generation of relevant data. Most of the currently available tools, although developed for use in LMICs may not be applicable to both countries, due to unique human resource and health system challenges.

In countries where infectious diseases, including bacterial infections, remain a major cause of morbidity and mortality, vis a viz an increasing burden of AMR, it is imperative that we implement to understand the drivers of AMR. Proper AMU surveillance systems will inform efforts toward proper access to antibiotics to treat infectious diseases and provide critical information to support optimal use to combat the emergence and spread of AMR. The recommendations made in this article should support the development of a strong sustainable national AMU surveillance program for both Tanzania and Uganda and other LMICs. Supporting the implementation of these recommendations will enable the country to progress on the WHO Benchmark 3.4, capacity 3, and 4 outlined above [12].

## Data Availability

Not applicable.
